# Antimicrobial Peptides from Amphibian Innate Immune System as Potent Antidiabetic Agents: A Literature Review and Bioinformatics Analysis

**DOI:** 10.1155/2021/2894722

**Published:** 2021-06-29

**Authors:** Hossein Soltaninejad, Hadi Zare-Zardini, Mahtab Ordooei, Yaser Ghelmani, Akram Ghadiri-Anari, Sanaz Mojahedi, Amir Ali Hamidieh

**Affiliations:** ^1^Department of Nanobiotechnology, Faculty of Biological Sciences, Tarbiat Modares University, Tehran, Iran; ^2^Medical Nanotechnology and Tissue Engineering Research Center, Yazd Reproductive Sciences Institute, Shahid Sadoughi University of Medical Sciences, Yazd, Iran; ^3^Hematology and Oncology Research Center, Shahid Sadoughi University of Medical Sciences, Yazd, Iran; ^4^Department of Pediatrics, Shahid Sadoughi University of Medical Sciences, Yazd, Iran; ^5^Clinical Research Development Center of Shahid Sadoughi Hospital, Shahid Sadoughi University of Medical Sciences, Yazd, Iran; ^6^Department of Internal Medicine, Diabetes Research Center, Shahid Sadoughi University of Medical Sciences, Yazd, Iran; ^7^Department of Biology, Faculty of Sciences, Science and Arts University, Yazd, Iran; ^8^Pediatric Cell and Gene Therapy Research Center, Tehran University of Medical Sciences, Tehran, Iran

## Abstract

Antimicrobial peptides, as an important member of the innate immune system, have various biological activities in addition to antimicrobial activity. There are some AMPs with antidiabetic activity, especially those isolated from amphibians. These peptides can induce insulin release via different mechanisms based on peptide type. In this review study, we collected all reported AMPs with antidiabetic activity. We also analyze the sequence and structure of these peptides for evaluation of sequence and structure effect on their antidiabetic activity. Based on this review, the biggest peptide family with antidiabetic activity is *temporins* with nine antidiabetic peptides. Frogs are the most abundant source of antidiabetic peptides. Bioinformatics analysis showed that an increase of positive net charge and a decrease of hydrophobicity can improve the insulinotropic effect of peptides. Peptides with higher positive net charge and Boman index showed higher activity. Based on this review article, AMPs with antidiabetic activity, especially those isolated from amphibians, can be used as novel antidiabetic drug for type 2 diabetes disease. So, amphibians are potential sources for active peptides which merit further evaluation as novel insulin secretagogues. However, strategy for the increase of stability and positive activity as well as the decrease of negative side effects must be considered.

## 1. Introduction

Diabetes, as a metabolic disorder, is characterized by high blood sugar level. Types 1 and 2 are the two main types of this disorder. Between these two types, type 2 diabetes is more common than type 1 [1, 2]. Type 2 diabetes, as a chronic disease, is characterized by high levels of blood sugar. The prevalence of this disease is increasing in the world due to lifestyle changes [2, 3]. Development of effective strategies for treatment and management of type 2 diabetes is necessary [4]. Glucose-lowering agents with natural origin can be considered for this treatment and management [5, 6]. Antimicrobial peptides are shown as important members of animal innate immune systems with broad antimicrobial activities. In addition to antimicrobial activity, these interesting peptides have various biological activities such as antiendotoxin, antiparasitic, anticancer, wound healing, spermicidal, insecticidal, and antioxidant [7–11]. The skins of amphibians produce peptides with inhibitory effects on bacterial and fungal growth [11]. These peptides were secreted in response to stress and infection. These peptides act as the first defense system against pathogens [12]. One of the interesting activities of some of these peptides is antidiabetic activity [13]. Antidiabetic peptides were first reported and identified from skin secretions of amphibians. These peptides have the ability to stimulate insulin release in vitro from BRIN-BD11 rat clonal *β* cells at low concentrations with low cell toxicity. Approximately, more than of 99% of antidiabetic peptides have been isolated from skin secretion of amphibians, especially from the species of *Anura*. In this review article, we assessed all reported antidiabetic peptides in terms of source, sequence, structure, and mechanism. We also evaluated these peptides for prediction and design of new drugs for the improvement of diabetic symptoms.

## 2. Methods

All qualitative and quantitative original articles with English language about antidiabetic peptides were entered. Important databases including Iran Medex, MEDLINE/PubMed, Google Scholar, and CINAHL and other pertinent references on websites were reviewed for article selection. The used search profiles were as follows: peptide/anti-diabetic/amphibain, AMPs/anti-diabetic/amphibain, peptide/antidiabetic/amphibain, AMPs/antidiabetic/amphibain, peptide/diabet/amphibain, AMPs/diabet/amphibain, peptide/anti-diabetic/frog, AMPs/anti-diabetic/frog, peptide/antidiabetic/frog, AMPs/antidiabetic/frog, peptide/diabet/frog, AMPs/diabet/frog, peptide/anti-diabetic/toad, AMPs/anti-diabetic/toad, peptide/antidiabetic/toad, AMPs/antidiabetic/toad, peptide/diabet/toad, AMPs/diabet/toad.

Articles not directly relevant to the subject were excluded. The EndNote software was used to handle the proper references.

For bioinformatics analysis (amino acid composition analysis, alignment, structure prediction, etc.) of acquired peptides, the CLC software was used.

## 3. Results and Discussion

Forty-seven AMPs with antidiabetic activity were acquired by database search. Among these peptides, only two peptides were identified from insects, social wasp, *Agelaia pallipes pallipes*. Other peptides were isolated from different amphibians. The properties of these 45 peptides are summarized in Table 1.

### 3.1. Brevinin-1CBb, Brevinin-1Pa, Brevinin-1E, Brevinin-2GUb, and Brevinin-2EC

These brevinin peptides showed a significant effect on the enhancement of insulin release at a concentration of 100 nM on rat BRIN-BD11 clonal beta cell line. These peptides had no effect on intracellular calcium. These peptides have weak hemolytic activity on human erythrocytes. The proposed mechanism for the antidiabetic effect of these peptides is possible involvement of both cyclic AMP-protein kinase A- and C-dependent G-protein sensitive pathways. The insulinotropic effect of these peptides has also been attributed to their effect on Rho G proteins [14–17].

### 3.2. Esculentin-1, Esculentin-1b, and Esculentin-2Cha

Similar to brevinin peptides, most of esculentin peptides showed significant insulin-releasing activity. Cyclic AMP-protein kinase A- and C-dependent G-protein sensitive pathways have been proposed for the antidiabetic action of esculentin peptides [16, 18].

### 3.3. Temporin-DRa, Temporin-DRb, Temporin-Oe, Temporin-CBa, Temporin-Va, Temporin-Vb, Temporin-Vc, Temporin-CBf, and Temporin-TGb

Various temporins, with different amphibian sources, showed significant stimulatory effects on insulin release from clonal rat BRIN-BD11 cells. These peptides had no effect on the release of lactate dehydrogenase. Temporin-Oe and Temporin-TGb also showed significant toxicity at optimal concentration for stimulation of insulin release. These peptides had no effect on intracellular calcium. The proposed mechanism for these peptides is KATP channel-independent pathway [17, 19].

### 3.4. Ranatuerin-1CBa, Ranatuerin-2CBc, and Ranatuerin-2CBd

These peptides were isolated from bullfrog *Lithobates catesbeianus*. These peptides can stimulate the release of insulin from the rat BRIN-BD11 clonal *β* cell line. Among these peptides, Ranatuerin-2CBd showed the highest stimulation of insulin release. On the other hand, among these peptides, Ranatuerin-2CBd is more cytotoxic than other peptides at optimal concentration. Based on studies, these three peptides have no effect on lactate dehydrogenase. This status indicated that these peptides preserve the integrity of the plasma membrane [17].

### 3.5. Dermaseptin B4 and Dermaseptin-LI1

Dermaseptins are considered as multifunctional AMPs. In between, Dermaseptin B4 and Dermaseptin-LI1 showed antidiabetic activity. Dermaseptin B4 and Dermaseptin-LI1 were isolated from *Phyllomedusa trinitatis* and *Agalychnis litodryas*, respectively. In glucose-responsive BRIN-BD11 cells, both peptides led to the stimulation of insulin release. The possible mechanism for induction of insulin secretion by these peptides has not been determined [20, 21].

### 3.6. Bombesin and Bombesin-Related Peptides

Bombesin is an AMP that is isolated from toad, *Bombina variegata*. Three bombesin and bombesin-related peptides with antidiabetic activity were isolated from *Bombina variegate*. The proposed mechanism of antidiabetic activity of bombesin and bombesin-related peptides is involvement of a cAMP-dependent, G protein-insensitive pathway [22].

### 3.7. Xenopsin and Xenopsin-AM2

Xenopsin and xenopsin-AM2 (containing the substitution Lys (3) ⟶ Arg in xenopsin) were isolated from *X. laevis* and *X. amieti* secretions, respectively. These two peptides lead to significant stimulations of insulin release from the rat BRIN-BD11 clonal *β* cell line. These peptides have no effect on the release of lactate dehydrogenase [23].

### 3.8. Palustrin-2CBa and Palustrin-1c

Palustrin-2CBa and Palustrin-1c were identified from skin secretions of *Lithobates catesbeianus* and *Lithobates palustris*, respectively. These peptides lead to significant stimulation of insulin release from BRIN-BD11 cells at low concentration [17, 24].

### 3.9. Phylloseptin-L2

A member of the phylloseptin family, Phylloseptin-L2, has potent activity for the increase of insulin release from the rat BRIN-BD11 clonal beta cell line. This peptide did not stimulate the release of the cytosolic enzyme, lactate dehydrogenase. This activity was maintained in the absence of extracellular Ca(2+). The proposed mechanism for this peptide for insulin release is independent of primary involvement influx of Ca(2+) or closure of ATP-sensitive K(+) channels [25].

### 3.10. RK-13

RK-13 is a 13-amino-acid insulinotropic peptide isolated from skin secretions of *Agalychnis calcarifer*. This peptide stimulated insulin release in a dose-dependent, glucose-sensitive manner, exerting its effects through a cyclic AMP-protein kinase A pathway independent of pertussis toxin-sensitive G proteins [26].

### 3.11. Pseudin-2

Pseudin-2 is a cationic peptide that is isolated from the skin of *Pseudis paradoxa*. This peptide leads to insulin release from the BRIN-BD11 clonal beta cell line without an increase of lactate dehydrogenase release. There is a mechanism involving Ca^2+^-independent pathways for this action of peptides [27].

### 3.12. GM-14

GM-14 is an insulin tropic peptide that originated from *Bombina variegate*. This peptide increases the insulin release via involvement of a cAMP-dependent, G protein-insensitive pathway [22].

### 3.13. IN-21

IN-21 was known as an insulinotropic peptide isolated from skin secretions of *Bombina variegate*. This peptide increases the insulin release via involvement of a cAMP-dependent, G protein-insensitive pathway [22].

### 3.14. Ocellatin-L2

Ocellatin-L2 is a glycine-leucine-rich peptide. This peptide was identified from norepinephrine-stimulated skin secretions of *Leptodactylus laticeps*. In higher concentration than 1 mM, this peptide can induce significant increase of the rate of insulin release from rat clonal BRIN-BD11 beta cells without significant effects on lactate dehydrogenase [28].

### 3.15. Plasticin-L1

Plasticin-L1 is a glycine-leucine-rich peptide. This peptide has a potent stimulatory effect on insulin release from rat clonal BRIN-BD11 beta cells. Plasticin-L1 has no effect on lactate dehydrogenase [28].

### 3.16. Tigerinin-1R

Tigerinin-1R is a cyclic dodecapeptide isolated from skin secretion of *Hoplobatrachus rugulosus*. This peptide has low cytotoxicity and hemolytic activity. Tigerinin-1R could significantly stimulate the insulin release from BRIN-BD11 cells. A study in a mouse model showed that this peptide increases insulin release and improves glucose tolerance [29].

### 3.17. Caerulein-B1

A caerulein-related peptide, Caerulein-B1, was identified from skin secretion of *Xenopus borealis*. This peptide has an additional Gly amino acid in comparison with caerulein and caerulein-B2. This peptide showed potent effect on stimulations of insulin release from the rat BRIN-BD11 clonal *β* cell line at low concentration [23].

### 3.18. Amolopin

Amolopin is an AMP with the highest similarity to temporins and vespid chemotactic peptides. This peptide showed a stimulatory effect on insulin release in INS-1 cells. Evaluation of mechanism indicated that the stimulatory effect had no effect on the increase of the influx of Ca^2+^ [30].

### 3.19. Alyteserin-2a

Alyteserin-2a, a peptide isolated from *Alytes obstetricans*, has high antidiabetic activity and low toxicity on human blood cells. Membrane depolarization and increased intracellular Ca^2+^ concentration have been considered as involved mechanisms in stimulation of insulin release [31].

### 3.20. Magainin-AM1 and Magainin-AM2

Magainin-AM1 and Magainin-AM2 are two AMPS isolated from *Xenopus amieti*. These peptides have antidiabetic activity. The proposed mechanism for action of these peptides is involvement of cell membrane depolarization and increase in intracellular calcium concentration. Studies also showed that Magainin-AM1 and Magainin-AM2 produced a significant improvement in glucose tolerance, insulin sensitivity, and improved beta cell functions in HFD-fed mice [32, 33].

### 3.21. Hymenochirin-1B

Hymenochirin-1B has an antidiabetic effect in BRIN-BD 11 cells. This peptide leads to the stimulation of insulin release from the pancreatic beta cells by the KATP channel-independent pathway. This peptide can increase the plasma insulin level after intraperitoneal administration in HFD-fed mice [34].

**Figure 1:**
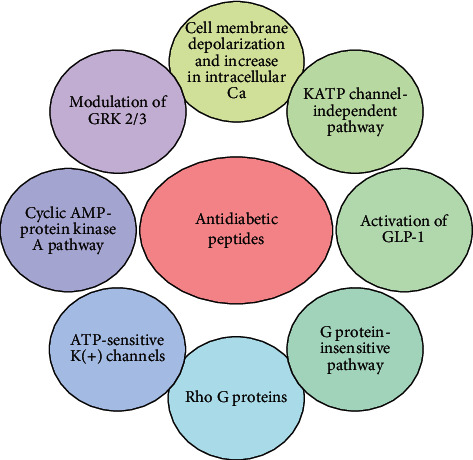
Proposed mechanisms of action for antidiabetic peptides.

Based on literature review, all proposed mechanisms of action for antidiabetic peptides have been summarized in Figure 1.

### 3.22. Pseudhymenochirin-2Pa and Pseudhymenochirin-1Pb

Pseudhymenochirin-1Pb and Pseudhymenochirin-2Pa are AMPs with stimulatory action on insulin release in BRIN-BD11 clonal *β* cells at low concentrations. The KATP channel-independent pathway is considered as the proposed mechanism for these peptides [35].

### 3.23. Statistical, Structural, and Bioinformatics Analysis

**Figure 2:**
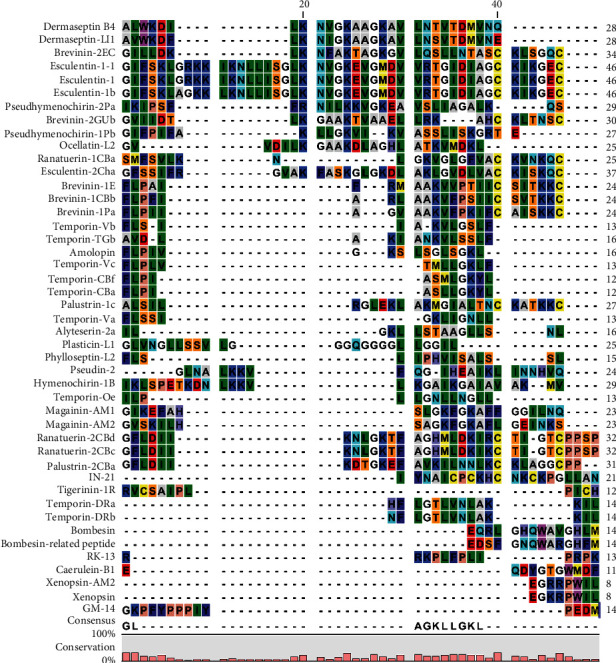
Sequence alignment of reported antidiabetic peptides.

The most abundant source of these peptides was the various species of frogs. The range of net charge for these 45 peptides is +2-6. Except for one peptide with 39% hydrophobicity, the other reported peptides have more than 48% hydrophobicity. The size range of these peptides is 8 to 46 amino acids. Among the *Rana* species, the most antidiabetic peptides were extracted from Asian frog *Hylarana guentheri*. Among the *Hylidae* species, four frog species have potent antidiabetic peptides: *Phyllomedusa trinitatis*, *Agalychnis calcarifer*, *Agalychnis litodryas*, and *Hylomantis lemur*. Among the *Bombinatoridae* species, six species of toads (genus *Bombina*) have potent peptides with insulin-releasing properties. In other species, the distribution of antidiabetic peptides does not follow a specific rule. Short half-life, stimulation of immune system, hemolysis, and toxicity are limitations for peptide use as antidiabetic agents. For example, Ocellatin-L2 has high hemolytic activity in addition to high activity in stimulating insulin release [28]. The change of amino acid content can lead to improved peptide ability. Related study showed that increase of positive net charge can increase the insulin releasing potency [25]. However, this change of net charge does not always lead to the same result. For example, increasing the net charge of peptide Pseudin-2 had no significant stimulatory effect on insulin release [27]. On the other hand, hydrophobicity is the most important property of antimicrobial peptides; studies showed that the reduction of hydrophobicity had no effect on insulinotropic effect of peptide, but decrease the hemolytic activity. So the decrease of hydrophobicity can be considered as a potent strategy for reduction of hemolytic activity without change of the antidiabetic activity of peptides [36]. Among all reported antidiabetic peptides, Magainin-AM2, with the lowest hydrophobicity (39%), showed the lowest hemolytic activity. In some peptides, *α*-amidation in the C-terminal region leads to the enhancement of antidiabetic potency. This *α*-amidation improves possible mechanism of action including peptide-involved membrane depolarization and an increase in intracellular Ca^2+^ concentration [37]. Owolabi et al. indicated that an increase of positive net charge of AMPs by adding cationic amino acids improved the potency of the insulinotropic activity [34]. This group also showed that an increase of hydrophobicity leads to a decrease of potency of the insulinotropic activity. The average length of all 45 peptides is 27.81 residues. The average net charge of all 45 peptides is 3.50. In Figure 2, the sequence alignment of the reported antidiabetic peptides is shown. Based on this figure, Ala, Gly, Lys, and Leu are consensus amino acids in all peptides. Bioinformatics analysis also showed that Leu, Gly, and Ala have the highest frequency in all reported antidiabetic peptides. The frequency of aromatic amino acids (Trp, Tyr, and Phe), His, Met, and Cys is low in these 45 peptides (Table 2). The Boman index is defined as the sum of the solubility values for all amino acids in sequence of peptide. This index predicts the potential of a peptide to bind to other proteins. The Boman index that is higher than 2.48 indicated high binding potential. Among the reported antidiabetic AMPs, peptides with higher Boman index have higher antidiabetic activity. This relationship is unknown. But it may be due to higher peptide ability to binding involved enzymes in insulin secretion and glucose uptake. Another strategy for the enhancement of half-life of antidiabetic peptides is substitution of L-amino acid with D-amino acid. Due to the small sizes and rapid elimination via the kidney, this strategy does not do well to solve the limitation of the peptide use [38]. Protection of peptide with various polymer coatings can be used for the enhancement of the therapeutic index of antidiabetic peptides [39]. This method is considered as a successful strategy for the improvement of peptide action, especially in the increase of their stability and reduction of their immunogenicity [40]. Increase of stability is related to the increase of peptide sizes that reduce rapid renal clearance. Conjugation with polyethylene glycol (PEGylation) [41, 42], anionic polypeptide-XTEN (864 amino acid-peptides containing A, E, G, P, S, and T) [43, 44], and hyaluronic acid (HAylation) [45, 46] are mostly used for physical shielding of antidiabetic peptides. Covalent and noncovalent interaction of peptide with serum albumins is also considered for modification of antidiabetic peptides [47–49].

### 3.24. Animal Studies

There are some studies about antidiabetic evaluation of the mentioned AMPs in animal models. Evaluation of these studies shows that all articles used low concentrations of peptides (nmol/kg body weight) in animal models [50, 51]. The literature review showed that the significant toxicity of AMPs (IC50) can occur in higher concentrations (micromole/kg body weight) [10, 52, 53]. So these tested peptides had antidiabetic activity at concentrations that are not toxic to the cells and animal model. However, based on in vivo studies, the abovementioned AMPs had low in vivo activity due to short blood circulation time. Increase of plasma stability and blood circulation time can improve the in vivo activity of AMPs. Physical shielding and attachment of albumin or antibody are the two main proposed strategies for the increase of plasma stability and blood circulation time of AMPs [54].

## 4. Conclusions

Based on this review study, antimicrobial peptides that originated from skin secretions of frogs and toads can stimulate insulin release. These natural peptides may be useful for type 2 diabetes treatment as complementary drugs. For improvement of antidiabetic activity, increase of half-life, reduction of toxicity on blood cell and other normal cells, change of net charge and hydrophobicity via change of amino acid composition, physical shielding of peptide with various polymers, and covalent or noncovalent conjugation with albumin can be considered as a model for preparation of synthetic peptides with antidiabetic activity. On the other hand, these natural peptides can be considered as a model for preparation of synthetic peptides with antidiabetic activity.

## Figures and Tables

**Table 1:** All reported antidiabetic peptides from amphibians.

Name	Source	Sequence	Net charge	Hydrophobic residue (%)	Boman index	3D structure
Brevinin-1CBb	*Lithobates catesbeianus*	FLPFIARLAAKVFPSIICSVTKKC	+4	62	-0.36	Unknown
Brevinin-1Pa	*Rana pipiens*	FLPIIAGVAAKVFPKIFCAISKKC	+4	66	-1.11	Unknown
Brevinin-1E	*Rana esculenta*	FLPAIFRMAAKVVPTIICSITKKC	+4	62	-0.36	Unknown
Brevinin-2GUb	*Hylarana guentheri*	GVIIDTLKGAAKTVAAELLRKAHCKLTNSC	+4	50	0.79	Unknown
Brevinin-2EC	*Pelophylax esculentus*	GILLDKLKNFAKTAGKGVLQSLLNTASCKLSGQC	+4	44	0.53	Unknown
Esculentin-1	*Rana esculenta*	GIFSKLGRKKIKNLLISGLKNVGKEVGMDVVRTGIDIAGCKIKGEC	+6	41	0.98	Helix
Esculentin-1b	*Rana esculenta*	GIFSKLAGKKLKNLLISGLKNVGKEVGMDVVRTGIDIAGCKIKGEC	+5	43	0.62	Unknown
Esculentin-2Cha	*Lithobates chiricahuensis*	GFSSIFRGVAKFASKGLGKDLAKLGVDLVACKISKQC	+5	48	0.53	Unknown
Temporin-DRa	*Rana draytonii*	HFLGTLVNLAKKIL	+2	57	-0.67	Unknown
Temporin-DRb	*Rana draytonii*	NFLGTLVNLAKKIL	+2	57	-0.53	Unknown
Temporin-Oe	*Rana ornativentris*	ILPLLGNLLNGLL	0	61	-2.15	Unknown
Temporin-TGb	*Rana tagoi*	AVDLAKIANKVLSSLF	+1	62	-0.18	Unknown
Temporin-Va	*Lithobates virgatipes*	FLSSIGKLIGNLL	+1	53	-1.18	Unknown
Temporin-Vb	*Lithobates virgatipes*	FLSIIAKVLGSLF	+1	53	-1.18	Unknown
Temporin-Vc	*Lithobates virgatipes*	FLPLVTMLLGKLF	+1	69	-2.29	Unknown
Temporin-CBf	*Lithobates catesbeianus*	FLPIASMLGKYL	+1	58	-1.55	Unknown
Temporin-CBa	*Lithobates catesbeianus*	FLPIASLLGKYL	+1	58	-1.77	Unknown
Ranatuerin-1CBa	*Lithobates catesbeianus*	SMFSVLKNLGKVGLGFVACKVNKQC	+4	52	0.05	Unknown
Ranatuerin-2CBc	*Lithobates catesbeianus*	GFLDIIKNLGKTFAGHMLDKIKCTIGTCPPSP	+2	40	0.34	Unknown
Ranatuerin-2CBd	*Lithobates catesbeianus*	GFLDIIKNLGKTFAGHMLDKIRCTIGTCPPSP	+2	40	0.64	Unknown
Dermaseptin B4	*Phyllomedusa trinitatis*	ALWKDILKNVGKAAGKAVLNTVTDMVNQ	+2	50	0.73	Unknown
Dermaseptin-LI1	*Agalychnis litodryas*	AVWKDFLKNIGKAAGKAVLNSVTDMVNE	+1	50	0.87	Unknown
Bombesin	*Bombina variegate*	EQRLGHQWAVGHLM	0	42	1.42	Unknown
Bombesin-related peptide	*Bombina variegate*	EDSFGNQWARGHFM	0	35	2.59	Unknown
Xenopsin	*Xenopus amieti*	EGKRPWIL	+1	37	1.77	Unknown
Xenopsin-AM2	*Xenopus amieti*	EGRRPWIL	+1	37	2.94	Unknown
Palustrin-2CBa	*Lithobates catesbeianus*	GFLDIIKDTGKEFAVKILNNLKCKLAGGCPP	+2	45	0.43	Unknown
Palustrin-1c	*Lithobates palustris*	ALSILRGLEKLAKMGIALTNCKATKKC	+5	51	0.59	Helix
Phylloseptin-L2	*Hylomantis lemur*	FLSLIPHVISALSSL	0	60	-1.33	Helix
RK-13	*Agalychnis calcarifer*	RRKPLFPLIPRPK	+5	30	2.93	Unknown
Pseudin-2	*Pseudis paradoxa*	GLNALKKVFQGIHEAIKLINNHVQ	+2	45	0.73	Unknown
GM-14	*Bombina variegate*	GKPFYPPPIYPEDM	-1	21	0.72	Unknown
IN-21	*Bombina variegate*	IYNAICPCKHCNKCKPGLLAN	+3	47	0.57	Helix
Ocellatin-L2	*Leptodactylus laticeps*	GVVDILKGAAKDLAGHLATKVMDKL	+1	52	0.25	Unknown
Plasticin-L1	*Leptodactylus laticeps*	GLVNGLLSSVLGGGQGGGGLLGGIL	0	40	-1.55	Unknown
Tigerinin-1R	*Hoplobatrachus rugulosus*	RVCSAIPLPICH	+1	58	-0.01	Unknown
Caerulein-B1	*Xenopus borealis*	EQDY(SO3)GTGWMDF	-3	27	2.08	Unknown
Amolopin	*Amolops loloensis*	FLPIVGKSLSGLSGKL	+3	43	-0.82	Unknown
Alyteserin-2a	*Alytes obstetricans*	ILGKLLSTAAGLLSNL	+2	56	-1.14	Unknown
Magainin-AM1	*Xenopus amieti*	GIKEFAHSLGKFGKAFFGGILNQ	+3	43	0.16	Unknown
Magainin-AM2	*Xenopus amieti*	GVSKILHSAGKFGKAFLGEINKS	+4	39	0.58	Unknown
Hymenochirin-1B	*Hymenochirus boettgeri*	IKLSPETKDNLKKVLKGAIKGAIAVAKMV	+6	48	0.47	Unknown
Pseudhymenochirin-2Pa	*Pseudhymenochirus merlini*	IKIPSFFRNILKKVGKEAVSLIAGALKQS	+5	48	0.55	Helix
Pseudhymenochirin-1Pb	*Pseudhymenochirus merlini*	GIFPIFAKLLGKVIKVASSLISKGRTE	+4	48	0.06	Helix

**Table 2:** The sequence information of reported antidiabetic peptides.

Amino acid	%	Average length	Average net charge
I	8.98	22.24	3.50
V	7.19		
L	13.93		
F	3.82		
C	2.69		
M	1.12		
A	10.11		
W	0.44		
G	11.68		
P	1.34		
T	2.92		
S	6.51		
Y	0		
Q	1.57		
N	4.26		
E	2.24		
D	2.69		
H	0.89		
K	15.73		
R	1.79		
